# PD-1H Expression Associated With CD68 Macrophage Marker Confers an Immune-Activated Microenvironment and Favorable Overall Survival in Human Esophageal Squamous Cell Carcinoma

**DOI:** 10.3389/fmolb.2021.777370

**Published:** 2021-12-07

**Authors:** Yuangui Chen, Rui Feng, Bailin He, Jun Wang, Na Xian, Gangxiong Huang, Qiuyu Zhang

**Affiliations:** ^1^ Department of Immunology, School of Basic Medical Sciences, Fujian Medical University, Fuzhou, China; ^2^ Department of Radiation Oncology, Fujian Medical University Union Hospital, Fuzhou, China; ^3^ Institute of Immunotherapy, Fujian Medical University, Fuzhou, China; ^4^ Department of Oncology, Fujian Medical University Union Hospital, Fuzhou, China; ^5^ Zhongshan School of Medicine, Sun Yat-Sen University, Guangzhou, China

**Keywords:** esophageal squamous cell carcinoma, tumor microenvironment, CD68 + myeloid cells, programmed death-1 homolog, prognosis

## Abstract

Esophageal squamous cell carcinoma (ESCC) is the most common type of esophageal carcinoma (EC) in China. Although the PD-1 inhibitor pembrolizumab has been approved to treat patients with EC, its therapeutic efficacy is limited. Thus, additional immunotherapeutic targets for EC treatment are needed. Programmed Death-1 Homolog (PD-1H) is a negative checkpoint regulator that inhibits antitumor immune responses. Here, PD-1H expression in 114 patients with ESCC was evaluated by immunohistochemistry. Next, 12 randomly selected tumor tissue sections were used to assess the colocalization of PD-1H protein and multiple immune markers by multiplex immunohistochemistry. Our results demonstrated that PD-1H was expressed at high frequency in ESCC tumor tissues (85.1%). PD-1H protein was predominantly expressed in CD68^+^ tumor-associated macrophages and expressed at low levels in CD4^+^ T cells and CD8^+^ T cells in ESCC tumor tissues. Furthermore, based on ESCC data in The Cancer Genome Atlas (TCGA), the gene expression levels of PD-1H were positively associated with the infiltration levels of immune-activated cells especially CD8^+^ cytotoxic T cells. In contrast, the gene expression levels of PD-1H were negatively correlated with myeloid-derived suppressor cells (MDSCs). Importantly, PD-1H expression in tumor sites was significantly correlated with favorable overall survival in patients with ESCC. Collectively, our findings first provided direct information on the PD-1H expression pattern and distribution in ESCC, and positive correlation of PD-1H expression with overall survival suggested PD-1H expression levels could be a significant prognostic indicator for patients with ESCC. Future studies need to explore the immunoregulatory of PD-1H in the tumor microenvironment of ESCC.

## Introduction

Esophageal carcinoma (EC) is one of the most fatal diseases worldwide and is the fourth most common cause of cancer-related death in China (Chen et al., 2016; Feng et al., 2019). Unlike North America and Europe, esophageal squamous cell carcinoma (ESCC) is the predominant subtype of esophageal carcinoma (EC) in China, accounting for approximately 90% of all patients with EC (Zeng et al., 2016). Targeting immune checkpoints has been demonstrated as a promising strategy in EC. Pembrolizumab, a PD-1-blocking checkpoint immunotherapy, was approved for advanced, PD-L1-positive ECs by the United States Food and Drug Administration (FDA) in 2017; however, only a small proportion of patients achieved therapeutic response (Kojima et al., 2020). Further exploration of novel immunotherapeutic targets for ESCC is needed. Programmed Death-1 Homolog (PD-1H), also known as V-domain Immunoglobulin Suppressor of T-cell activation (VISTA), DD1α, c10orf54, Dies1, or Gi24, is a novel T-cell cosignaling molecule. Previous studies demonstrated that the immuneregulatory pathways for PD-1 and PD-1H are functionally nonredundant by using PD-1H and programmed death-1 (PD-1) knockout mice (Liu et al., 2015). PD-1H can function as both a receptor in T lymphocytes and a ligand on antigen-presenting cells (Bharaj et al., 2014; Flies et al., 2014; Le Mercier et al., 2014; Lines et al., 2014a). Moreover, PD-1H was highly expressed on CD11b^+^ myeloid cells and macrophages and was expressed at low levels on T cells, including CD4^+^ T cells, CD8^+^ effector T cells, and Foxp3^+^ Tregs (Bharaj et al., 2014; Le Mercier et al., 2014). PD-1H showed constitutive expression characteristics (Lines et al., 2014b). Considering different expression patterns of PD-1H and its nonredundant activities compared to other immune checkpoint regulators, PD-1H has become a promising target of cancer immunotherapy [11].

Recently, PD-1H was reported to be highly expressed in various human cancers, including prostate cancer (Gao et al., 2017), non-small cell lung cancer (Villarroel-Espindola et al., 2018), colorectal carcinoma (Xie et al., 2018), ovarian and endometrial cancer (Mulati et al., 2019), esophageal adenocarcinoma (Loeser et al., 2019), and epithelioid malignant pleural mesothelioma (Muller et al., 2020); however, limited data about PD-H expression in ESCC have been reported. Here, we analyzed the PD-1H expression pattern in tumor microenvironment of 114 patients with ESCC and its correlation with the infiltrating number of T cells and myeloid cells. Next, the relationships between *PD-1H* mRNA levels and the infiltration of various immune cells were assessed in ESCC based on The Cancer Genome Atlas (TCGA) database. We further investigated the correlation of PD-1H expression with the survival outcomes of ESCC patients.

## Materials and Methods

### Patients and Sample Preparation

Tumor tissues were obtained from 114 patients with ESCC who underwent surgery at Fujian Medical University Union Hospital from 2015 to 2016. None of these patients received preoperative radiotherapy or chemotherapy before surgery. A retrospective review of the medical records of these patients was performed between January 1, 2015 and June 30, 2020. Relevant clinical data were collected by retrospective review of the files of the patients, and follow-up data were available for all patients. Peripheral blood mononuclear cells (PBMCs) were isolated from six patients with ESCC using Ficoll-Paque PLUS (#171140, GE Healthcare, United States) according to the manufacturer’s instructions. All procedures followed the national and institutional ethical standards, and all samples were obtained in accordance with the institutional policies. All protocols were reviewed and approved by the Research Ethics Committee of Fujian Medical University Union Hospital (No. 2020ky085).

### Immunohistochemistry

Tumor tissues were fixed in formaldehyde and paraffin-embedded following standard procedures. Tissue sections were incubated with PD-1H antibodies (1:200, #64953, Cell Signaling Technology) and then immunostained using the MaxVision™ HRP-Polymer anti-Rabbit IHC Kit (KIT-5005, Maixin Biol, Fuzhou, China) following the manufacturer’s instructions. Immunostaining of PD-1H was microscopically evaluated (Olympus BX53, Japan). During evaluation of immunostained sections, the investigators were blinded to the clinical status of the patients. After screening the whole section, 10 randomly selected microscopic fields were examined at × 200 magnification (×20 objective lens and ×10 ocular lens; 0.74 mm^2^ per field). Expression levels for PD-1H were scored semiquantitatively based on staining intensity and percentage of stained cells using the immunoreactive score (IRS) as described (Kakavand et al., 2015). The percentage of positively stained cells (PP) was assigned as a numerical score: 0, negative; 1, <25%; 2, 26–50%; 3, 51–75%; and 4, >75% positive cells. The intensity (SI) of the immunostained areas was defined as follows: 0, negative; 1, weak (+); 2, moderate (++); and 3, strong (+++). An immunoreactive score (IRS) ranging from 0 to 12 was calculated using the following formula: IRS = PP × SI. The expression of PD-1H was defined as follows: 0–1, negative; >1–4, mild; >4–8, moderate; >8–12, strong. Based on this formula, the average expression score for each patient was calculated, and the score with the maximum split in survival was chosen as the cutoff using the cutpoint function of Evaluate Cutpoints software (R survMisc package) as described (Ogluszka et al., 2019). Then, the patients were classified into high- or low-expression score groups by a 7.2 score.

### Multiplex Immunohistochemistry

Multiplex immunohistochemistry (mIHC) was performed by staining 4-μm-thick formalin-fixed, paraffin-embedded whole-tissue sections with multiple primary antibodies and a TSA 5-color fluorescent IHC kit (Yuanxi Bio, China) as described in a previous study (Stack et al., 2014). The concentration and order of the four antibodies were optimized, and the spectral library was built based on the single-stained slides. Four primary antibody/fluorophore pairs were applied in order: anti-CD4 antibody (#YX32005, Yuanxi Bio)/NEON-TSA 520, anti-CD8 (#YX63005, Yuanxi Bio)/NEON-TSA 620, anti-CD68 (#GM087602, Gene Tech)/NEON-TSA 670, and anti-PD-1H (#64953, Cell Signaling Technology)/NEON-TSA 570. Twelve randomly selected tumor tissue sections were used for mIHC. The slides were deparaffined with a graded series of xylene, and dehydrated in a gradient of alcohols, and then antigen retrieval was performed by microwave. After incubation with 3% H_2_O_2_ for 10 min, the tissues were blocked in blocking buffer for another 10 min at room temperature. Then, the tissues were incubated with primary antibodies, followed by secondary-HRP and TSA working solutions, respectively, according to the manufacturer’s instructions. Finally, the slides were mounted with ProLong Gold Antifade Reagent with DAPI (#P36931, Invitrogen). All slides were scanned using an Aperio Versa 8 tissue imaging system (Leica, Germany). Images were analyzed using HALO image analysis software (Indica Lab, United States).

### Acquisition of PD-1H Expression Profiles From TCGA Datasets

RNA sequencing (RNA-Seq) data and clinical information for 182 esophageal carcinoma patients (TCGA ESCC) were collected and downloaded through the TCGA data portal (https://portal.gdc.cancer.gov/). The transcript expression levels were estimated using the fragments per kilobase per million fragments mapped (FPKM) method in HTSeq. Kaplan–Meier survival curve analysis was used to analyze survival between the PD-1H ^high^ and PD-1H ^low^ groups as previously described (Rich et al., 2010). Immune cell infiltration was evaluated using Cell-type Identification By Estimating Relative Subsets Of RNA Transcripts (CIBERSORT) algorithm (Newman et al., 2015) based on Tumor Immune Estimation Resource (TIMER) (Li et al., 2020) as described. The gene expression data with standard annotation were uploaded to the CIBERSORT web portal (http://cibersort.stanford.edu/). The algorithm was run by 1,000 permutations and the LM22 signature. Samples with CIBERSORT *p* < 0.05 were considered eligible for subsequent analysis. The correlation between PD-1H expression and immune infiltration patterns was estimated using linear regression analysis.

### Flow Cytometry

Human peripheral blood mononuclear cells (PBMCs) were isolated from the whole blood of six patients with ESCC, and then T cells and monocytes were enriched by Rosettesep™ Human T Cell Enrichment Cocktail and Human Monocyte Enrichment Cocktail (#15028_c, Stemcell Technologies, United States), respectively. T cells and monocytes were stained with antibodies against human CD3e (#560352, BD Biosciences), CD4 (#300532, BioLegend), CD8a (#300922, BD Biolegend), CD14 (#301807, BioLegend), PD-1H (#566672, BD Biosciences), and matched isotype controls (#555749, BD Biosciences) at different time points after blood collection (6, 24, and 48 h). PBMCs, purified T cells, and purified monocytes were stimulated with PMA (50 ng/ml, #P1585, Sigma-Aldrich), LPS (200 ng/ml, #L4391, Sigma-Aldrich), and polyI:C (2 µg/ml, #P0913, Sigma-Aldrich), and anti-CD3/anti-CD28 Dynabeads (#11132D, Gibco) for 24 h. T cells were treated with different concentrations of polyhydroxyalkanoate (PHA) (#PZ0135, Sigma-Aldrich) for 5 h. Samples were run on a BD FACSVerse™ (BD Biosciences, United States) and analyzed using FlowJo software version 10 (BD Biosciences, United States).

### Quantitative Real-Time PCR

Human T cells were stimulated with different concentrations of PHA for 5 h. Total RNA of T cells was extracted using TRIzol (#15596-026, Invitrogen) and was reverse-transcribed into cDNA using a One Step TB Green® PrimeScript PLUS RT-PCR Kit (#RR096A, Takara Bio, United States) according to the manufacturer’s instructions. Quantitative real-time polymerase chain reaction (qRT-PCR) was performed with SYBR™ Green PCR Master Mix (#4309155, Invitrogen). PD-1H primer sequences were as follows: forward (5′-ACG​CCG​TAT​TCC​CTG​TAT​GTC-3′) and reverse (5′-TTG​TAG​AAG​GTC​ACA​TCG​TGC-3′). The expression levels of *PD-1H* were normalized to the expression of GAPDH. The relative expression levels of *PD-1H* mRNA in treated cells were calculated using their expression in control cells as a reference transcript.

### Statistical Analysis

All statistical analyses were performed using GraphPad Prism 8.0 (GraphPad, Canada). All data are shown as the mean ± SD unless otherwise stated. The chi-squared and Fisher’s exact tests were used for interdependence between staining and clinical data. Univariate and multivariate analyses were performed for prognostic factors of overall survival with the Cox regression model, and the Kaplan–Meier method and the log-rank test were used for survival curves with a plot. *p* < 0.05 was considered statistically significant. The results represent at least two experiments unless otherwise stated.

## Results

### PD-1H Protein Was Highly Expressed in ESCC Tumor Tissues

**FIGURE 1:**
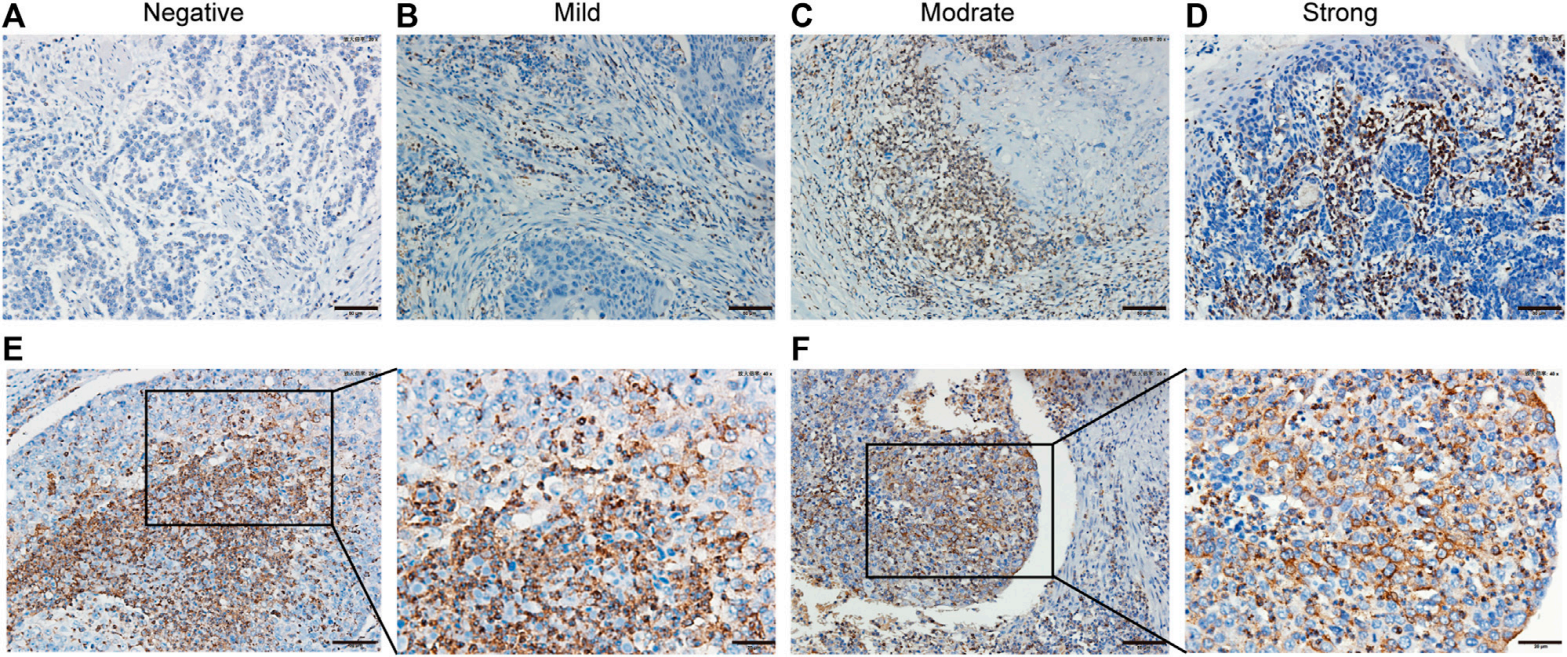
PD-1H expression in ESCC analyzed by immunohistochemistry. **(A–D)** Immunohistochemistry staining of PD-1H in ESCC tumor tissues was performed and calculated using immunoreactive score (IRS), which was categorized as 0–1 (negative), >1–4 (mild), >4–8 (moderate), or >8–12 (strong). Representative images of four categories of classification of PD-1H expression in ESCC tumor tissues are shown. **(E–F)** Two representative cases with strong positive PD-1H staining demonstrated that PD-1H protein was predominantly located in the membrane and cytoplasm of tumor cells and immune cells. Scale bars: 50 μm, original magnification × 200; 25 μm, original magnification ×400. PD-1H: Programmed Ddeath-1 Homolog; ESCC: esophageal squamous cell carcinoma. Scale bar: 25 μm, original magnification × 400.

The clinical pathological features of 114 patients with ESCC are listed in Table 1. A total of 85.1% tumor tissues (97/114 cases of ESCC) were positive for PD-1H expression by IHC staining. According to the IRS-classification scoring systems, predominant expression evaluation was moderate positive staining (46.5%, 53/114) followed by mild positive staining (23.7%, 27/114), strong positive staining (14.9%, 17/114) and negative staining (14.9%, 17/114), and the representative images of four categories of classification are shown in Figures 1A–D. Figures 1E,F indicated that PD-1H protein was predominantly located in the membrane and cytoplasm of positive staining cells. Next, the optimal cutoff for “low” and “high” expression was determined by the inbuilt algorithm in the survMisc R package. A score of 7.2 was used as an optimal cutoff point and all patients were divided into a high-expression group (74.5%, 85/114) and a low-expression group (25.5%, 29/114). Then, the relationships of PD-1H expression level and clinical characteristics of ESCC were investigated. Our results showed that PD-1H expression level was negatively associated with lymph node metastasis (*p* = 0.020) and Union for International Cancer Control (UICC) stages (*p* = 0.009) (Table 1). However, the expression of PD-1H exhibited no correlation with sex, age, tumor stage, or tumor grades of differentiation.

**TABLE 1:** The correlation of PD-1H expression and clinicopathologic parameters in 114 patients with ESCC.

		*N*	%	PD-1H expression				
				Low	%	High	%	*p*-value
Sex	Female	32	28.1	23	71.8	9	28.1	0.811
	Male	82	71.9	62	75.6	20	24.3	
Age group	<65 years	85	74.5	63	74.1	22	25.8	1.000
	≥65 years	29	25.5	22	75.8	7	24.1	
Tumor stage	T1	25	21.9	15	60	10	40	0.287
	T2	21	18.4	16	76.2	5	23.8	
	T3	66	57.8	52	78.8	14	21.2	
	T4	2	1.7	2	100	0	0	
Lymph node metastasis	N0	51	44.7	31	60.8	20	39.2	0.020
	N1	35	30.7	30	85.7	5	24.3	
	N2	23	20.1	19	82.7	4	17.3	
	N3	5	4.3	5	100	0	0	
Grades of differentiation	G1	38	33.3	25	65.8	13	34.2	0.276
	G2	55	48.2	43	78.2	12	21.8	
	G3	9	7.8	8	88.8	1	11.2	
UICC stage	I	27	23.7	17	63	10	37	0.009
	II	37	32.4	23	62.2	14	37.8	
	III	44	38.6	39	88.6	5	11.4	
	IV	6	5.3	6	100	0	0	

UICC, union for international cancer control; PD-1H, Programmed death-1 homolog; ESCC, esophageal squamous cell carcinoma.

### PD-1H Was Predominantly Expressed in CD68^+^ Myeloid Cells of the Tumor Immune Microenvironment

**FIGURE 2:**
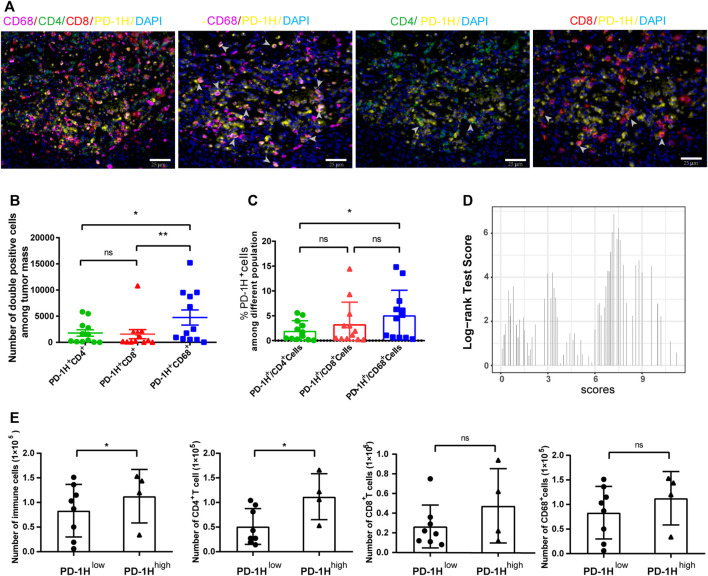
PD-1H expression in tumor-infiltrating immune cells assessed by multiplex immunohistochemistry (mIHC). **(A)** Representative images of mIHC staining of PD-1H, CD4, CD8, CD68, and DAPI in ESCC tumor tissues. Scale bar: 25 μm, original magnification × 400. **(B)** The cell numbers of PD-1H^+^CD68^+^ myeloid cells were significantly higher than cell numbers of PD-1H^+^CD4^+^ and PD-1H^+^CD8^+^ cells. **(C)** The percentage of PD-1H positive cells among CD4^+^ T cells, CD8^+^ T cells, and CD68^+^ cells was estimated. **(D)** The expression score of PD-1H was calculated and classified into high-expression (PD-1H ^high^) or low-expression (PD-1H ^low^) groups by using the R package of “survMisc”. **(E)** The absolute counts of immune cells and CD4^+^ T cells in tumor sites were significantly higher in patients with high PD-1H expression than in patients with low PD-1H expression. ns = non-significant, *p < 0.05, **p < 0.01.

To further investigate cell types with PD-1H expression in the tumor immune microenvironment, we randomly selected 12 tumor sections from 114 ESCC patients for multiplexed immunohistochemistry. Colocalization staining of PD-1H and multiple immunohistochemical markers was measured in tumor tissues. Our results documented that the majority of the PD-1H protein were colocalized with the CD68 macrophage marker. In contrast, CD4 and CD8 colocalized with PD-1H to significantly lower levels, although some areas of colocalization could be found (Figure 2A). The Halo image analysis platform was used to quantify the density and colocalization of positively stained cells. The results showed that the amount of PD-1H colocalized with CD68 was significantly higher than that colocalized with the amount of PD-1H colocalized CD4 (*p* = 0.0069) or CD8 cells (*p* = 0.0115) (Figure 2B). Likewise, among PD-1H-positive cells, the percentage of CD68^+^ cells were significantly higher than the percentage of CD4^+^ cells (*p* = 0.0142) (Figure 2C). Consistent with this result, the absolute number of CD4^+^ T cells and total immune cells was higher in the high PD-1H expression group than in the low PD-1H expression group (Figures 2D,E).

### PD-1H mRNA Levels Were Correlated with Immune Cell Infiltration in ESCC

**FIGURE 3:**
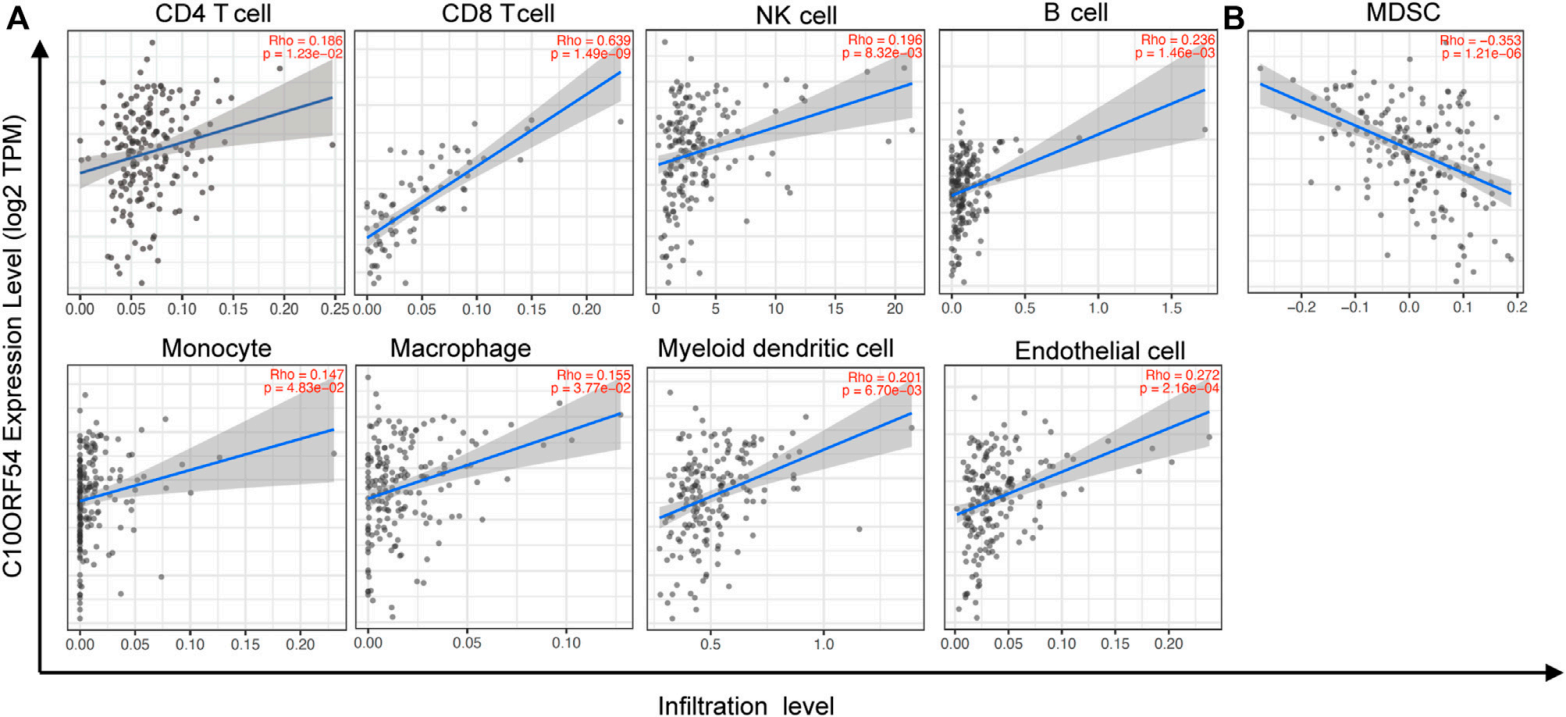
The relationships of PD-1H expression with immune cell infiltration were assessed in ESCC based on TCGA database. The CIBERSORT algorithm was used to identify the immune cell infiltration signatures. **(A)** Positive correlations of PD-1H with CD4^+^ T cells (Rho = 0.186, *p* = 0.012), CD8^+^ T cells (Rho = 0.639, *p* < 0.001), CD68^+^ macrophages (Rho = 0.155, *p* = 0.038), CD1c^+^ myeloid dendritic cells (Rho = 0.201, *p* = 0.007), CD14^+^ monocytes (Rho = 0.147, *p* = 0.048), CD56^+^ NK cells (Rho = 0.196, *p* = 0.008), CD20^+^ B cells (Rho = 0.236, *p* = 0.001), and CD31^+^ endothelial cells (Rho = 0.272, *p* < 0.001). **(B)** The negative correlation of PD-1H with CD11b^+^CD33^+^HLA-DR^−^MDSCs (Rho = −0.353, *p* < 0.001). CIBERSORT: Cell type Identification By Estimating Relative Subsets Of RNA Transcripts.

As our results indicated that PD-1H was highly expressed in CD68^+^ myeloid cells of human ESCC tumors, we next assessed possible relationships of *PD-1H* mRNA expression level and the infiltration of nine types of immune cells by searching ESCC datasets from TCGA. The data showed that *PD-1H* mRNA (C10orf54) expression levels were significantly positively correlated with cell infiltration of CD4^+^ T cells (Rho = 0.186, *p* = 0.012), CD8^+^ T cells (Rho = 0.639, *p* < 0.001), CD68^+^ macrophages (Rho = 0.155, *p* = 0.038), CD1c^+^ myeloid dendritic cells (Rho = 0.201, *p* = 0.007), CD14^+^ monocytes (Rho = 0.147, *p* = 0.048), CD56^+^ NK cells (Rho = 0.196, *p* = 0.008), CD19^+^ B cells (Rho = 0.236, *p* = 0.001), and CD31^+^ endothelial cells (Rho = 0.272, *p* < 0.001) (Figure 3A). In contrast, the *PD-1H* mRNA expression level was negatively correlated with CD11b^+^CD33^+^HLA-DR^−^ myeloid-derived suppressor cells (MDSCs) (Rho = −0.353 *p* < 0.001) (Figure 3B).

### PD-1H Protein Expression Level Was Correlated with Overall Survival of Patients with Human ESCC

**FIGURE 4:**
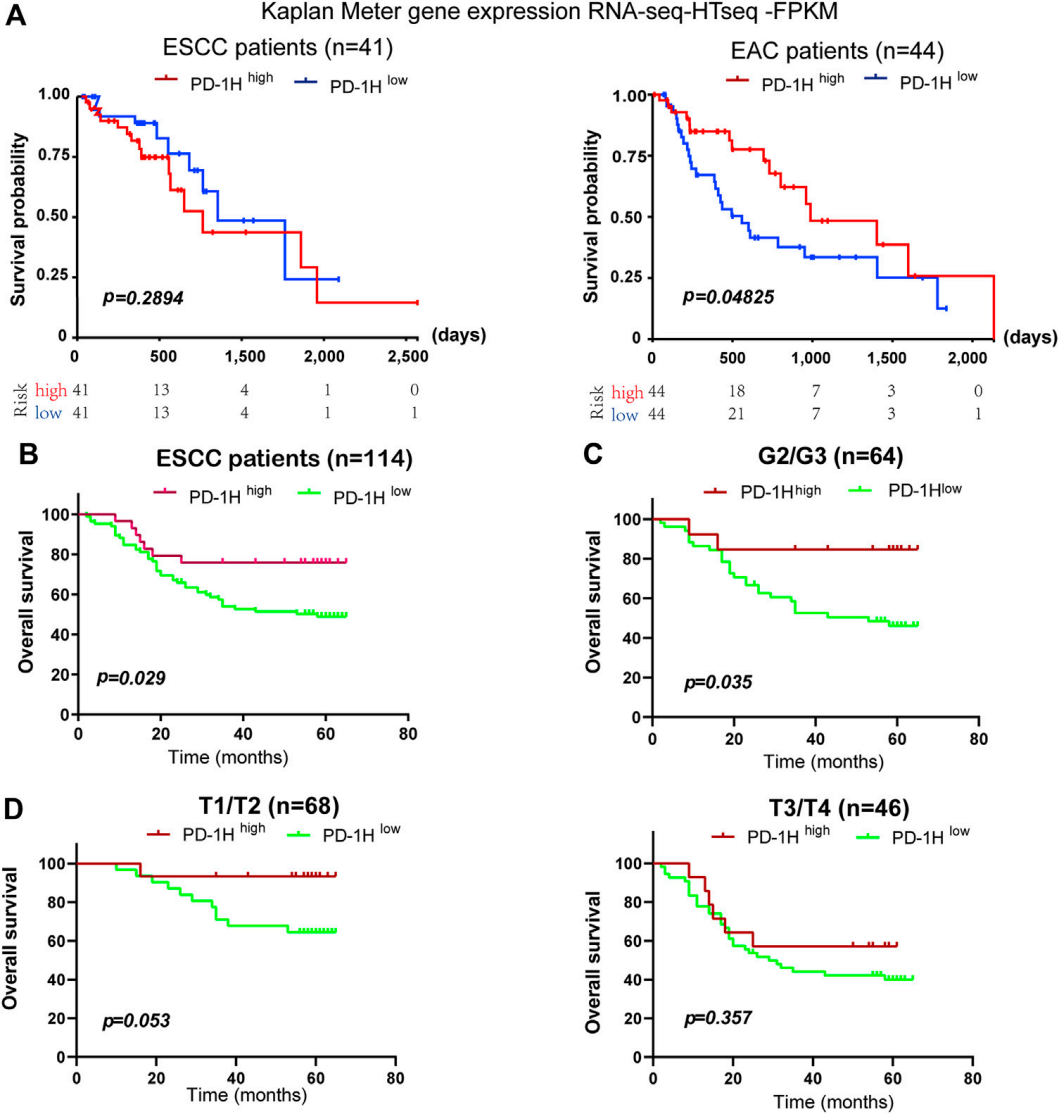
The association between PD-1H expression levels and overall survival in ESCC. **(A)** The association of *PD-1H* mRNA expression and overall survival of patients was assessed in 41 ESCC patients and 44 EAC patients from the TCGA database. **(B–D)** With the applied scoring system, a score of 7.2 as the optimal cutoff point was identified by the “survMisc” package in R. The correlation between PD-1H protein expression and prolonged overall survival in 114 ESCC patients **(B)**, in the G1 and G2/3 subgroups **(C)** and in the T1/T2 and T3/T4 tumor stage subgroups **(D)** was analyzed by using log-rank (Mantel-Cox) test in GraphPad 8.0. ESCC, esophageal squamous cell carcinoma; EAC, esophageal adenocarcinoma; G (G2/G3), Grades of differentiation; T (T1/T2, T3/T4), Tumor stage.

Recent studies demonstrate that high PD-1H expression correlates with a favorable prognosis in patients with colorectal cancer, high-grade serous ovarian cancer, triple-negative breast cancer, hepatocellular carcinoma, and esophageal adenocarcinoma (Zhang et al., 2018; Loeser et al., 2019; Cao et al., 2020; Zong et al., 2020; Zong et al., 2021). Here, 114 patients who were followed for 65 months were subjected to survival analysis, and the correlation of PD-1H expression with clinicopathologic parameters was analyzed by Kaplan–Meier survival and Cox regression models (Tables 2, 3). In the validation cohort, univariate Kaplan–Meier analyses showed that tumor stage (*p* = 0.001), lymph node metastasis (*p* = 0.0 02), UICC stage (*p* = 0.001), and PD-1H expression (*p* = 0.029) were associated with the overall survival of patients with human ESCC. However, only tumor stage remained significant on multivariate Cox regression analysis. Although no statistically significant association was found between *PD-1H* mRNA expression and overall survival in ESCC from TCGA (Figure 4A), Kaplan–Meier analyses showed that ESCC patients with high PD-1H protein expression had significantly improved overall survival (HR = 0.60; 95% CI, 0.27–0.93; *p* = 0.029; Figure 4B) in the exploratory cohort, especially among the G2/3 subgroup (Figures 4C,D). These results indicated that the PD-1H expression level is positively associated with a favorable prognosis in patients with ESCC.

**TABLE 2:** Univariate Kaplan–Meier analyses of prognostic parameters in 114 patients with ESCC.

	—	*N*	95% CI		
			Lower	Upper	*p*-value
Sex	Female	32	44.344	59.210	0.048
	Male	82	37.524	47.943	
Age group	<65 years	85	41.353	51.432	0.357
	≥65 years	29	33.415	50.281	
Tumor stage	T1 or T2	46	50.219	60.281	0.001
	T3 or T4	68	32.621	44.444	
Lymph node metastasis	N0	51	47.522	58.686	0.002
	Nx	63	33.024	44.976	
Grades of differentiation	G1 or G2	93	39.684	49.429	0.979
	G3	9	26.669	56.183	
UICC stage	I or II	64	47.030	56.931	0.001
	III or IV	50	29.825	43.627	
PD-1H expression	Low	85	37.550	47.429	0.029
	High	29	45.389	60.823	

CI, confidence interval; HR, hazard ratio; UICC, union for international cancer control; PD-1H, programmed death-1 homolog; ESCC, esophageal squamous cell carcinoma.

**TABLE 3:** Multivariate analyses for the prediction of overall survival in patients with ESCC.

	HR	95% CI		
		Lower	Upper	*p*-value
Sex (female vs. male)	0.679	0.342	1.351	0.270
Age group (<65 vs. ≥ 65 years)	1.997	0.901	4.426	0.089
Tumor stage (T1/2 vs. T3/4)	2.589	1.100	6.096	0.029
Lymph node metastasis (N0 vs. Nx)	1.97	0.766	5.068	0.16
Grades of differentiation (G1/2 vs. G3)	1.290	0.439	3.789	0.642
UICC stage (I/II vs. III/IV)	0.749	0.265	2.119	0.586
PD-1H expression (low vs. high)	0.637	0.260	1.402	0.240

CI, confidence interval; HR, hazard ratio; UICC, union for international cancer control; PD-1H, Programmed death-1 homolog; ESCC, esophageal squamous cell carcinoma.

### PD-1H Expression Was Detected in Activated T Cells and Peripheral Monocytes

**FIGURE 5:**
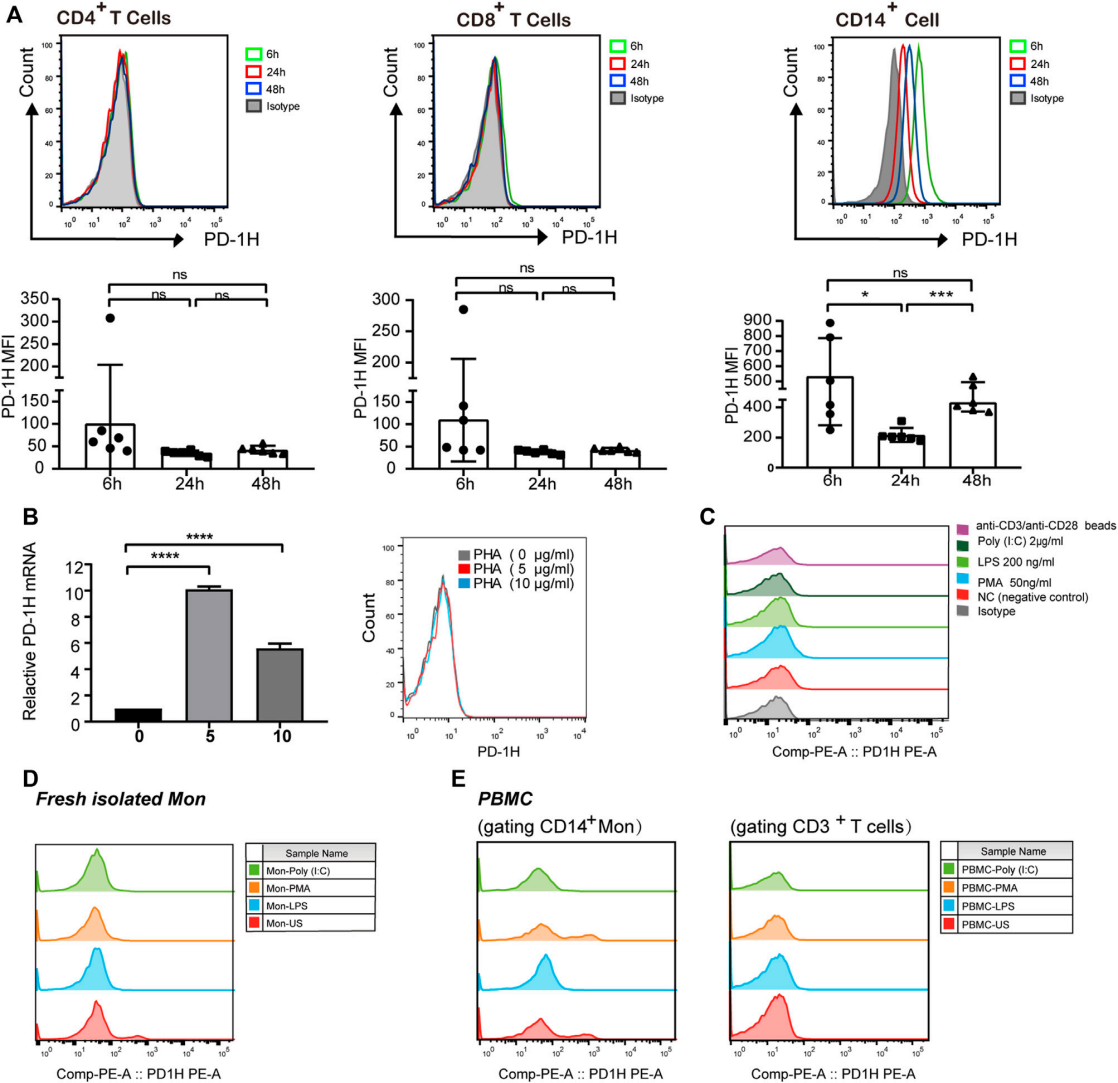
PD-1H expression in peripheral T cells and monocytes. **(A)** PD-1H expression on the surface of CD4^+^ T cells, CD8^+^ T cells, and CD14^**+**^ monocytes was analyzed by FCM at 6, 24, and 48 h after blood collection. The expression levels of PD-1H cells are shown in a representative FCM histogram (upper graph), and the mean fluorescence intensity (MFI) of PD-1H positive staining was pooled from two independent experiments (lower graph). **(B)** T cells were treated with different concentrations of PHA (0, 5, and 10 μg/ml), and then *PD-1H* mRNA and PD-1H cell surface expression was analyzed by qRT-PCR (left graph) and FCM (right graph), respectively. **(C)** Membrane PD-1H expression was analyzed 24 h after T cells were treated with PMA (50 ng/ml), LPS (200 ng/ml), poly I:C (2 μg/ml), or anti-CD3/anti-CD28 beads. **(D,E)** Membrane PD-1H expression was analyzed on the cell surface of purified monocytes and PBMCs after the cells were stimulated with PMA, LPS, and poly (I:C) for 24 h. The data are representative of at least two independent experiments. ns = non-significant, *p < 0.05, ***p < 0.001, ****p < 0.0001. FCM, flow cytometry; qRT-PCR, real-time quantitative reverse transcription PCR; PBMC, peripheral blood mononuclear cells; Mon, monocytes; NC, negative control; PHA, polyhydroxyalkanoates; LPS, lipopolysaccharide; poly (I:C), polyinosinic:polycytidylic acid; PMA, Phorbol 12-myristate 13-acetate.

Given that high PD-1H expression was found in the tumor microenvironment of ESCC, we further examined PD-1H expression in peripheral T cells and monocytes derived from six ESCC patients by FCM and qRT-PCR. No positive staining was detected on the surface of CD4^+^ and CD8^+^ T cells PD-1H expression at 6, 24, and 48 h of blood collection. In contrast, PD-1H expression was found in CD14^+^ monocytes, and the mean fluorescence intensity (MFI) of PD-1H positive staining on monocytes at 24 and 48 h of blood collection was lower than MFI of PD-1H positive staining on monocytes at 6 h (Figure 5A). Although the expression levels of *PD-1H* mRNA in CD3^+^ T cells were upregulated by the stimulation with PHA for 24 h (Figure 5B), PD-1H protein on the surface of T cells was not detectable (Figure 5C). Moreover, no significant PD-1H expression was detected on the T cell surface after T cells were stimulated with different stimulators, including PMA, LPS, poly (I:C), and TCR signaling (anti-CD3/anti-CD28 Dynabeads). PD-1H expression on CD14^+^ cells was transiently downregulated 24 h after isolation, and membrane PD-1H on CD14^+^ monocytes was decreased by the treatment with PMA, LPS, or poly (I:C) (Figure 5D). Intriguingly, PD-1H expression was maintained on CD14-positive cell surface after monocytes were mixed with T cells and treated with the PMA for 24 h**.** However, no detectable PD-1H membrane expression was found in activated T cells (Figure 5E).

## Discussion

Following the initial identification of the immune checkpoint receptors CTLA-4 and PD-1, other Ig superfamily T-cell inhibitory ligands/receptors, such as LAG-3, TIM-3, TIGIT, BTLA, B7-H3, and B7-H4 have been indicated as important regulators of antitumor immunity (Gavrieli et al., 2006; Yi and Chen, 2009; Anderson et al., 2016; Andrews et al., 2017; Janakiram et al., 2017). PD-1H belongs to the CD28 superfamily, sharing 24 and 32% sequence similarity with PD-L1 and PD-1 molecules, respectively (Flies et al., 2011; Wang et al., 2011). Unlike other immune checkpoint receptors that are induced on activated T lymphocytes, PD-1H was reported to be constitutively expressed on myeloid cells and naïve T cells, exerting both ligand and receptor functions (Lines et al., 2014a; Gao et al., 2017; ElTanbouly et al., 2020). Increasing evidence indicates that PD-1H has a different expression pattern in the tumor microenvironment of different tumor subtypes. Mulati et al. reported that PD-1H was highly expressed in tumor cells of human ovarian and endometrial cancers using tissue microarray analysis and immunohistochemical staining (Mulati et al., 2019). Several lines of evidence support that PD-1H was expressed predominantly in myeloid cells of multiple myeloma, melanoma, and colorectal carcinoma, which were assessed mainly by multiplex immunofluorescence (Choi et al., 2020; Mutsaers et al., 2021; Zong et al., 2021). PD-1H has also been reported to be constitutively expressed in naïve T cells by single-cell RNA and ATAC sequencing technologies (ElTanbouly et al., 2020). The discrepancy between these studies might be due to the different PD-1H antibodies and variable tumor samples used in the experiments. Recently, PD-1H was reported to be expressed with CD68 and CD4 in most cases of esophageal adenocarcinoma (EAC) and to have no reliable coexpression with CD8 (Loeser et al., 2019). To date, little is known about PD-1H expression in human squamous cell carcinoma (ESCC). In this study, our data demonstrated that PD-1H was expressed at a high frequency in tumor tissues of ESCC (85.1%, 97/114), as analyzed by immunohistochemical staining. Multiplex immunohistochemistry showed that PD-1H was detected in CD4 T cells, CD8 T cells, and CD68 macrophages of ESCC tumor tissues. PD-1H protein was diffusely distributed within the plasma membrane of tumor infiltrating immune cells, and PD-1H expression levels were significantly higher in CD68-positive macrophages than T cells. Next, the optimal cutoff for PD-1H expression levels was determined using the method implemented in the survMisc R package. Our data showed that the number of immune cells, especially CD4^+^ T cells, was significantly higher in ESCC cases with high levels of PD-1H expression than in ESCC cases with low levels of PD-1H. Altogether, our findings firstly provide direct information on PD-1H expression pattern and distribution in ESCC tumor tissues. ESCC and EAC have almost completely distinct geographic patterns, time trends, and primary risk factors (Abnet et al., 2018; Salem et al., 2018). In EAC, PD-1H was found to be coexpressed with CD68 and CD4; however, no reliable coexpression with CD8 was detectable. The different expression patterns of PD-1H between ESCC and EAC suggested the potentially different immune regulation mechanism of PD-1H in ESCC.

To explore the potential role of PD-1H in ESCC, we examined the association between the levels of *PD-1H* mRNA expression and the transcripts of various immune genes in ESCC datasets of 182 patients from TCGA. Intriguingly, high levels of *PD-1H* mRNA expression were significantly associated with stronger CD8^+^ cytotoxic T-cell tumor infiltration. A weaker positive association was found in CD4^+^ helper T cells, CD56^+^ NK cells, CD20^+^ B lymphocytes, CD14^+^ monocytes, CD68^+^ macrophages, CD1c^+^ myeloid dendritic cells, and CD31^+^ endothelial cells. Conversely, the *PD-1H* mRNA expression level was negatively correlated with myeloid-derived suppressor cells (MDSCs). Monocytes, macrophages, and MDSCs represent different stages of myeloid differentiation. MDSCs represent a phenotypically heterogeneous population of immature myeloid cells and can be divided into granulocytic (G-MDSCs) and monocytic (M-MDSCs) subpopulations. Macrophages are the most abundant tumor-infiltrating immune cells and display noticeable plasticity, which allows them to perform several functions within the tumor microenvironment (Kwak et al., 2020). PD-1H expression was observed in a range of tumor-infiltrating immune cells with inconsistent expression profiles in different types of cancers. Most previous studies supported that PD-1H is a negative immune regulator. PD-1H/VISTA is highly expressed on MDSCs derived from patients with AML, and *PD-1H* gene knockdown by siRNA potently reduces the MDSC-mediated inhibition of CD8 T-cell activity (Wang et al., 2018). In a B16 melanoma model, PD-1H blockade with antibodies decreased the infiltration of M-MDSCs in the tumor microenvironment through impairment of M-MDSC migration into tumor sites (Le Mercier et al., 2014). Broughton et al. found that PD-1H deficiency reduced the migration of *PD-1H* KO macrophages and MDSCs into the tumor microenvironment by altering chemokine receptor recycling (Broughton et al., 2019). Recently, Rogers et al. reported that agonistic PD-1H antibodies promoted human monocytes to express HLA, CD80, and CD40 in an Fc effector functional manner, indicating that PD-1H can serve as an activating receptor on human monocytes (Rogers et al., 2021). Our findings showed that the *PD-1H* mRNA expression level was positively correlated with the infiltration of CD8^+^ T cells, CD4^+^ T cells, and CD68^+^ macrophages, whereas the *PD-1H* mRNA expression level was negatively correlated with HLA-DR^−^CD33^+^ MDSC infiltration. Based on our findings, it is tempting to speculate that high levels of PD-1H expression associated with an immune-activated microenvironment contribute favorable clinical outcomes in human esophageal squamous cell carcinoma.

Increasing evidence suggests a correlation between PD-1H expression and tumor patient prognosis. Boger et al. reported that PD-1H expression did not correlate with prognosis in patients with gastric cancer (Boger et al., 2017). Nonetheless, a higher level expression of PD-1H combined with low CD8 expression in tumor microenvironment is associated with poor prognosis and lymph node metastases in patients with oral squamous cell cancer (Wu et al., 2017). In contrast, higher expression of PD-1H in the tumor compartment predicted longer overall survival in patients with nonsmall cell lung cancer (Villarroel-Espindola et al., 2018), high-grade serous ovarian cancer (Zong et al., 2020), colorectal carcinoma (Zong et al., 2021), hepatocellular carcinoma (Zhang et al., 2018), triple-negative breast cancer (Cao et al., 2020), hepatocellular carcinoma, and epithelioid malignant pleural mesothelioma (Muller et al., 2020). Loeser et al. also demonstrated an improved median overall survival in EAC patients with PD-1H expression compared to those without PD-1H expression, especially in the pT1/2 subgroup in EAC (Loeser et al., 2019). Consistent with most previous reports, our results showed that PD-1H expression was positively correlated with favorable overall survival of ESCC patients, especially in the G2/G3 subgroup. Nevertheless, there was no significant association between *PD-1H* mRNA expression and overall survival of patients with esophageal cell carcinoma from TCGA database. These paradoxical results might be due to the different tumor samples used in the experiments. Given that “mRNA expression” is always inconsistent with “protein expression” for multiple immune regulators, we further analyzed *PD-1H* mRNA and surface protein expression in peripheral T cells and monocytes. PD-1H membrane-bound expression was positive in CD14^+^ monocytes derived from ESCC patients, whereas no detectable surface expression was found in CD4^+^ and CD8^+^ T cells. Intriguingly, the PD-1H MFI on the cell surface was decreased after monocyte isolation, indicating that PD-1H expression was potentially dependent on cell–cell interactions *in vivo*. PD-1H was reported to be constitutively expressed on cell surface of naïve CD4^+^ and CD8^+^ T cells, and could be further upregulated by PMA plus ionomycin (a non-specific activator of T cells) (Flies et al., 2011). Our findings demonstrated that membrane PD-1H expression was still detected when monocytes were cocultured with PMA-activated T cells. Nevertheless, membrane PD-1H expression was undetectable on T cells by the stimulation of PHA, LPS, poly (I:C), or TCR signaling *in vitro*. These results indicated that PD-1H expression on the cell surface of monocytes was dependent on T-cell activation. The modulatory mechanism of the PD-1H protein in tumor microenvironment of ESCC remains unclear and worthwhile for further investigation.

In conclusion, our study demonstrated that PD-1H, which was predominantly expressed in CD68^+^ myeloid cells in ESCC tumor tissues, was positively associated with the infiltration levels of immune-activated cells. Importantly, high levels of PD-1H expression were correlated with favorable overall survival in patients with ESCC, suggesting that PD-1H protein could be a prognostic indicator for patients with ESCC. Moreover, T-cell activation by nonspecific agents was beneficial to maintain PD-1H expression on the cell surface of monocytes. Additional studies are warranted to understand the role and regulatory mechanism of PD-1H in the tumor microenvironment of ESCC.

## Data Availability

The raw data supporting the conclusion of this article will be made available by the authors, without undue reservation.
